# The staining effect of different mouthwashes containing nanoparticles on dental enamel

**DOI:** 10.4317/jced.52199

**Published:** 2015-10-01

**Authors:** Neda Eslami, Farzaneh Ahrari, Omid Rajabi, Roya Zamani

**Affiliations:** 1 DDS, MS, Assistant Professor of Orthodontics, Dental Research Center, School of Dentistry, Mashhad University of Medical Sciences, Mashhad, Iran; 2 DDS, MS, Associate Professor, Department of Drug and Food Control, School of Pharmacy, Mashhad University of Medical Sciences, Mashhad, Iran; 3 DDS, School of Dentistry, Mashhad University of Medical Sciences, Mashhad, Iran

## Abstract

**Background:**

This study aimed to evaluate the effects of several mouthwashes containing nanoparticles on discoloration of dental enamel, and compare the results with that of 0.2% chlorhexidine (CHX).

**Material and Methods:**

Sixty intact premolars were randomly assigned to six groups. A spectrophotometer was used to measure the color of the teeth (T1) according to the CIELAB system. The specimens in groups 1 to 4 were then immersed in colloidal solutions containing nanoTiO2 (Group 1), nanoZnO (Group 2), nanoAg (Group 3) and nanoCuO (Group 4). In groups 5 and 6, a 0.2% CHX mouthwash and distilled water were used as positive and negative controls, respectively. After 24 hours of immersion, color determination was repeated (T2). The third color assessment was accomplished after brushing (T3). The L, a, and b values were recorded and the color change (?E) between different stages was calculated.

**Results:**

ANOVA revealed significant between-group differences in the color change between T1 and T2 stages, as well as between T1 and T3 time points (*p*<0.05), whereas the color change between T2 and T3 was not significantly different among the study groups (*p*=0.09). ?ET1-T3 was significantly lower in the specimens immersed in distilled water or CHX as compared to the nanoparticle-containing mouthwashes (*p*<0.05). The highest ?E value pertained to the specimens immersed in nanoZnO-containing solution. The TiO2 nanoparticles caused the lowest staining among the tested nanoparticles.

**Conclusions:**

The mouthwashes containing nanoparticles produced comparable or even greater enamel discoloration compared to CHX. Brushing had little effect on removal of induced stains.

** Key words:**Nanoparticle, mouthrinse, mouthwash, staining, enamel, discoloration, chlorhexidine.

## Introduction

Dental caries and periodontal problems are the most common oral diseases throughout the world (1). Although there are several strategies to prevent or reduce these problems, the mechanical methods of plaque control including tooth brushing and application of interproximal cleaners are still considered as the golden standard in this aspect. However, in disabled or traumatized patients as well as those underwent oral surgery, the effective plaque control may not be possible with mechanical approaches. Furthermore, some patients, such as those undergoing fixed orthodontic therapy, exhibit a great susceptibility to caries formation and periodontal problems (2-5). In these situations, the use of mouthwashes as disinfection solutions could be considered as a supplementary method for controlling dental plaque. However, the side effects of some mouthwashes such as staining of enamel and restorative materials and calculus formation (6-8) have limited their application for oral hygiene measures. Therefore, it is of great interest to find mouthwashes that provide effective plaque control with minimal side effects.

Recently, nanotechnology has been employed in dentistry to provide materials with enhanced mechanical properties and antibacterial effects (9,10). It is believed that the antibacterial property is due to the decreased size of the nanoparticles which increases their surface contact area and thus their interaction with organic and inorganic molecules (11). The colloidal solutions containing metal nanoparticles were prepared in a previous study (11) and their bactericidal and bacteriostatic properties were evaluated against cariogenic and periodontal disease bacteria. It was concluded that the nanoTiO2 containing mouthwash could be considered as an alternative to chlorhexidine (CHX), provided the lack of other side effects such as cytotoxicity or discoloration of enamel and restorative materials (11).

There is little information regarding the staining effect of nanoparticle-containing solutions when applied as mouthwashes on dental enamel. The discoloration of tooth and restorative materials by CHX has been demonstrated in several studies (7,8). The present investigation aimed to evaluate the staining effects of several mouthwashes containing titanium dioxide nanoparticles (nanoTiO2), zinc oxide nanoparticles (nanoZnO), silver nanoparticles (nanoAg) and copper oxide nanoparticles (nanoCuO) on dental enamel, and compare the results with that of 0.2% CHX. Furthermore, the ability of brushing to eliminate the discoloration produced by these mouthrinses was investigated.

## Material and Methods

-Preparation of colloidal solutions containing nanoparticles

NanoTiO2, nanoZnO, nanoAg and nanoCuO were purchased from PlasmaChem GmbH (Berlin, Germany). According to the supplier, nanoparticles had more than 99% purity after ignition. The nanoparticles were added to a water-base solution in Rese-arch Laboratory, School of Pharmacy, Mashhad University of Medical Sciences, Mashhad, Iran. The colloidal solutions containing nanoparticles were prepared with an initial concentration of 25 ppm and were autoclave sterilized before the experiments. The particle size analysis was performed to assess the size and the distribution of nanoparticles. The average size of the nanoparticles ranged from 40-60 nm for nanoTiO2 and nanoCuO, 50-60 nm for nanoAg and 25 nm for nanoZnO.

-Color change measurement

Sixty upper premolars extracted for orthodontic reasons were gathered and stored in distilled water at room temperature until the time of the experiment. The teeth were intact and without any caries or structural defects. The sample was randomly allocated into six groups of 10 each.

Before the colorimetric measurements, the teeth were cleaned with water slurry of pumice and rubber prophylactic cups, rinsed with tap water, and then immersed for 24 hours in distilled water at 37°C. The Easyshade spectrophotometer (Vita Zahnfabrik, Bad Säckingen, Germany) was used to assess the color of buccal enamel surface (T1, baseline examination) according to the CIELAB (Commission Internationale de l’Eclairage L*a* and b*) color space system. In this system, the L coordinate refers to the value or degree of lightness, whereas the a and b values indicate positions on red/green (+a=red, -a=green) and yellow/blue (+b=yellow, -b= blue) axes, respectively.

After baseline color examination, the specimens in groups 1 to 4 were exposed to colloidal solutions containing nanoTiO2 (Group 1), nanoZnO (Group 2), nanoAg (Group 3) and nanoCuO (Group 4). In groups 5 and 6, a 0.2% chlorhexidine mouthwash (CHX) and distilled water were used as positive and negative controls, respectively. The immersion period was 24 hours during which the solutions were shaken every 3 hours to create homogeneity. The teeth were then rinsed with water for 1 minute, dried with cotton rolls, and color determination was repeated (T2, after immersion in mouthwash).

Finally, the teeth were subjected to a brushing procedure in which an Oral B CrossAction power toothbrush and Crest toothpaste were employed under a standardized load. Each tooth was brushed for 1 minute, then rinsed with water, dried with cotton rolls, and subjected to color measurement to determine the L, a, and b values again (T3, after brushing).

The color measurements were performed twice and the mean values of L, a, and b were determined for each specimen. The color change (?E) between the different treatment stages was calculated using the following formula: ?E= [(?a)2 +(?b)2 +(?L)2]0.5.

-Statistical analysis

The normal distribution of the data was confirmed by the Kolmogorov-Smirnov test and the homogeneity of variances with the Levene’s test. One way analysis of variance (ANOVA) was run to compare the color change between the two measurements at T1 to T3 time points (?E) among the study groups, followed by Tukey multiple range test for pairwise comparisons. The statistical analysis was performed using Statistical Package for the Social Sciences (version 16.0, SPSS Inc, Chicago, Ill) and the significance level was determined at *p*<0.05.

## Results

 Table 1 demonstrates the descriptive statistics and the results of statistical analysis regarding the color change (?E) between the different treatment stages for the six groups. As seen in  Table 1, the highest ?E value pertained to the specimens immersed in nanoZnO-containing solution, whereas the lowest one was observed in those soaked in distilled water. Brushing the specimens was not effective in reducing the stains caused by immersion in different media.

**Table 1:**
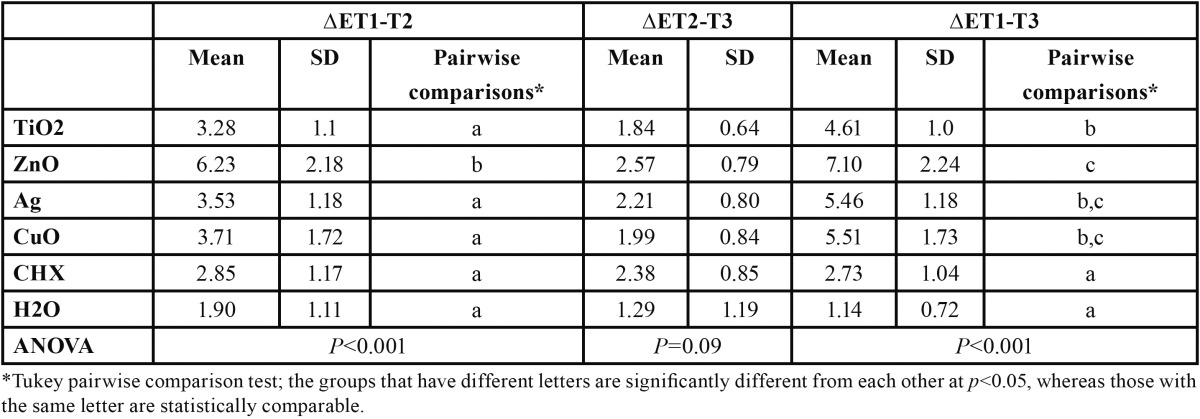
The mean, standard deviation (SD) and the results of statistical analysis regarding the color change between different stages among the experimental groups.

One way analysis of variance revealed significant between-group differences in the color change between T1 (baseline) and T2 (after immersion in mouthwash) stages, as well as between T1 and T3 (after brushing) time points ( Table 1). The experimental groups, however, did not exhibit any significant difference in the color change between T2 and T3 stages ( Table 1).

Pairwise comparisons by Tukey test revealed that ?ET1-T2 was significantly greater in group 2 (nanoZnO-containing solution) than the other study groups (*p*<0.05;  Table 1), which were not significantly different from each other. Tukey test also revealed that the specimens immersed in distilled water or CHX exhibited significantly lower color change between T1 and T3 stages as compared to the nanoparticle-containing mouthwashes (*p*<0.05;  Table 1). Furthermore, ?ET1-T3 was significantly lower in nanoTiO2-containing group compared to that of the nanoZnO group (*p*<0.05), whereas both groups showed comparable color change to nanoAg- and nanoCuO-containing solutions ( Table 1).

## Discussion

The appearance of the dentition is of great concern to a large number of people seeking dental treatments. Tooth discolorations are usually unsightly especially in anterior areas of the mouth. The limitations of the mechanical methods of plaque control have drawn the researchers’ attention to the application of different mouthrinses, which may be nevertheless associated with major side effects such as enamel staining.

In the current study, Easy-Shade spectrophotometer was used for color assessment of dental enamel. Color differences can be measured using spectrophotometers or colorimeters (12). These instruments reduce the subjective errors of color assessment with naked eyes, which cannot quantitatively assess slight color differences (13). According to Llena, *et al.* (14), Easy-Shade has high reproducibility and can be used for tooth color measurements. CIE lab color system was developed by the Commission International d’Eclairage for measuring colors based on human perception, and it is widely used for color assessments. In this system, ?E (color difference value) shows the relative color change between repeated color measurements (12,15).

The present study investigated enamel color changes caused by mouthwashes containing nanoTiO2, nanoZnO, nanoAg and NanoCuO and compared the results with that of CHX. After baseline color measurement (T1), the teeth were immersed in different mouthwashes for 24 hours, and color assessment was repeated (T2). Other studies also immersed the specimens for 12 or 24 hours in different media (15-17). The third color measurement was performed after brushing (T3). This was done to assess the effect of brushing on attenuating any stain that has been produced by the solutions. The results of this study indicated varying degrees of enamel discoloration after immersion in CHX and nanoparticle-containing mouthwashes. The NanoZnO-containing solution resulted in the most severe color change, whereas the staining effects of other mouthwashes were comparable to each other. Brushing had little effect on removal of stains induced by mouthwashes, and no significant difference was found regarding the color difference before and after brushing among the study groups.

When the color change between T1 and T3 stages was considered, it was found that the teeth immersed in nanoZnO solution exhibited the most sever color change, followed by nanoCuO, nanoAg, nanoTiO2 and CHX, respectively. This indicates that nanoZnO is not suitable to be inserted in the oral solutions, whereas nanoTiO2 should be further investigated for this application, as it caused the least amount of discoloration compared to the other nanoparticles. It should be noted that the nanoparticle-containing mouthwashes used in this study produced stains with greater severity than that of CHX; therefore, they cannot be considered, in the current form, as suitable alternatives to eliminate the side effects of CHX.

Chlorhexidine (CHX) is routinely prescribed because of its strong anti-microbial activity. It affects a wide range of bacteria, candida, and some spices of viruses including HIV and Hepatitis virus (8,18,19). Furthermore, CHX can prevent the formation and accumulation of bacterial plaque and the development of gingivitis (6,7,20,21). However, frequent use of CHX may have detrimental effects on oral and dental tissues. CHX has been found to induce brown stains on enamel, composite restorations, oral mucosa, and tongue (6-8). The staining effect of CHX on dental enamel was also observed in the present study. Three mechanisms have been suggested for discoloration caused by CHX (22,23): A. non-enzymatic browning reactions (Maillard reactions), B. formation of pigmented (Fe, Sn)- sulphides, and C. reaction of dietary chromogens with CHX. Most evidence indicates that the likely cause of CHX-induced staining is the interaction or precipitation of anionic dietary chromogens with adsorbed cationic antiseptics (8,24). The severity of discoloration depends on the CHX concentration and its duration of application (18,25).

Since nanotechnology was introduced to dentistry, various applications for nanomaterials have been suggested (26-28). As new formulations appear, it would seem prudent to at least evaluate their properties by comparison with an established product. There is limited data available regarding the different properties of nanoparticle-containing mouthwashes. A recent study investigated the antibacterial effects of colloidal solutions containing nanoparticles against *Streptococcus mutans* and *Streptococcus sanguis* (11). The results showed that the nanoTiO2-containg solution resulted in the least number of *Streptococcus mutans* colonies compared to other nanoparticle-containing mouthwashes, and its antibacterial properties were comparable to that of 0.2% CHX (11). To the best of our knowledge, there has been no study comparing the staining potential of these newly developed mouthrinses. Therefore, direct comparison of the present findings with those of other investigations is not possible.

Um and Ruyter (29) reported that coffee-induced discoloration is not reduced by brushing, whereas discoloration caused by tea is usually cleaned after brushing. Ertas, *et al.* (15) reported that composite discoloration induced by tea is due to adsorption of polar colorants onto the surface of resin composites, whereas coffee-induced discolorations are due to both adsorption and absorption of polar colorants onto the surface of materials, which may prevent their removal by brushing. Considering the nanosize of the particles used in the mouthwashes in this study, their deep penetration into the surface irregularities could contribute to the resistance of the stains to brushing. Further studies are warranted to elucidate the mechanism of tooth discoloration induced by nanoparticle-containing mouthwashes.

In the CIE lab color system, ?E values greater than 3.3 units are clinically perceptible (30). ?L is more important compared to ?a, and ?b parameters because its changes can be detected more easily by the human eyes. In the present study, ?ET1-T3 was greater than 3.3 units in nanoparticle-containing groups, and thus it was clinically detectable. However, the nanoparticle-containing mouthwashes were prepared with the initial concentration of 25 ppm, which was several times greater than their MIC (Minimum Inhibitory Concentration) and MBC (Minimum Bactericidal Concentration) against common bacteria in the oral cavity (11). Therefore, it is expected that less discoloration would be observed after clinical application of these mouthwashes at their effective dose. It should be noted that in vitro studies may not provide a reliable simulation of the clinical situations. The staining effect of nanoparticle-containing mouthwashes in the presence of saliva and their possible interactions should be further clarified in future studies.

## Conclusions

1- The mouthrinses containing metal nanoparticles produced the same or even greater enamel discoloration compared to that of CHX.

2- The TiO2 nanoparticle caused the lowest and the ZnO nanoparticle produced the greatest staining among the tested nanoparti-cles.

3- Brushing had little effect on removal of stains induced by mouthrinses.

## References

[1] Heravi F, Ahrari F, Mahdavi M, Basafa S (2014). Comparative evaluation of the effect of Er:YAG laser and low level laser irradiation combined with CPP-ACPF cream on treatment of enamel caries. J Clin Exp Dent.

[2] Lucchese A, Gherlone E (2013). Prevalence of white-spot lesions before and during orthodontic treatment with fixed appliances. Eur J Orthod.

[3] Julien KC, Buschang PH, Campbell PM (2013). Prevalence of white spot lesion formation during orthodontic treatment. Angle Orthod.

[4] Benson PE, Parkin N, Dyer F, Millett DT, Furness S, Germain P (2013). Fluorides for the prevention of early tooth decay (demineralised white lesions) during fixed brace treatment. Cochrane Database Syst Rev.

[5] Shahabi M, Ahrari F, Mohamadipour H, Moosavi H (2014). Microleakage and shear bond strength of orthodontc brackets bonded to hypomineralized enamel following different surface preparations. J Clin Exp Dent.

[6] Sheen S, Owens J, Addy M (2001). The effect of toothpaste on the propensity of chlorhexidine and cetyl pyridinium chloride to produce staining in vitro: a possible predictor of inactivation. J Clin Periodontol.

[7] Lorenz K, Bruhn G, Heumann C, Netuschil L, Brecx M, Hoffmann T (2006). Effect of two new chlorhexidine mouthrinses on the development of dental plaque, gingivitis, and discolouration. A randomized, investigator-blind, placebo-controlled, 3-week experimental gingivitis study. J Clin Periodontol.

[8] Zanatta FB, Antoniazzi RP, Rösing CK (2010). Staining and calculus formation after 0.12% chlorhexidine rinses in plaque-free and plaque covered surfaces: a randomized trial. J Appl Oral Sci.

[9] Heravi F, Ramezani M, Poosti M, Hosseini M, Shajiei A, Ahrari F (2013). In Vitro Cytotoxicity Assessment of an Orthodontic Composite Containing Titanium-dioxide Nano-particles. J Dent Res Dent Clin Dent Prospects.

[10] Sun J, Forster AM, Johnson PM, Eidelman N, Quinn G, Schumacher G (2011). Improving performance of dental resins by adding titanium dioxide nanoparticles. Dent Mater.

[11] Ahrari F, Eslami N, Rajabi O, Ghazvini K, Barati S (2015). The antimicrobial sensitivity of Streptococcus mutans and Streptococcus sangius to colloidal solutions of different nanoparticles applied as mouthwashes. Dent Res J (Isfahan).

[12] Joiner A (2004). Tooth colour: a review of the literature. J Dent.

[13] Jahanbin A, Basafa M, Moazzami M, Basafa B, Eslami N (2014). Color Stability of Enamel following Different Acid Etching and Color Exposure Times. J Dent Res Dent Clin Dent Prospects.

[14] Llena C, Lozano E, Amengual J, Forner L (2011). Reliability of two color selection devices in matching and measuring tooth color. J Contemp Dent Pract.

[15] Erta? E, Güler AU, Yücel AC, Köprülü H, Güler E (2006). Color stability of resin composites after immersion in different drinks. Dent Mater J.

[16] Celik C, Yuzugullu B, Erkut S, Yamanel K (2008). Effects of mouth rinses on color stability of resin composites. Eur J Dent.

[17] Guler AU, Yilmaz F, Kulunk T, Guler E, Kurt S (2005). Effects of different drinks on stainability of resin composite provisional restorative materials. J Prosthet Dent.

[18] Addy M, Wade W, Goodfield S (1991). Staining and antimicrobial properties in vitro of some chlorhexidine formulations. Clin Prev Dent.

[19] Torres SR, Peixoto CB, Caldas DM, Akiti T, Barreiros MG, de Uzeda M (2007). A prospective randomized trial to reduce oral Candida spp. colonization in patients with hyposalivation. Braz Oral Res.

[20] Brecx M, Macdonald LL, Legary K, Cheang M, Forgay MG (1993). Long-term effects of Meridol and chlorhexidine mouthrinses on plaque, gingivitis, staining, and bacterial vitality. J Dent Res.

[21] Lang NP, Hase JC, Grassi M, Hämmerle CH, Weigel C, Kelty E (1998). Plaque formation and gingivitis after supervised mouthrinsing with 0.2% delmopinol hydrochloride, 0.2% chlorhexidine digluconate and placebo for 6 months. Oral Dis.

[22] Ellingsen JE, Rolla G, Eriksen HM (1982). Extrinsic dental stain caused by chlorhexidine and other denaturing agents. J Clin Periodontol.

[23] Eriksen HM, Nordbo H, Kantanen H, Ellingsen JE (1985). Chemical plaque control and extrinsic tooth discoloration. A review of possible mechanisms. J Clin Periodontol.

[24] Addy M, Mahdavi SA, Loyn T (1995). Dietary staining in vitro by mouthrinses as a comparative measure of antiseptic activity and predictor of staining in vivo. J Dent.

[25] Hoffmann T, Bruhn G, Richter S, Netuschil L, Brecx M (2001). Clinical controlled study on plaque and gingivitis reduction under long-term use of low-dose chlorhexidine solutions in a population exhibiting good oral hygiene. Clin Oral Investig.

[26] Hannig M, Hannig C (2010). Nanomaterials in preventive dentistry. Nat Nanotechnol.

[27] Salata O (2004). Applications of nanoparticles in biology and medicine. J Nanobiotechnology.

[28] Kanaparthy R, Kanaparthy A (2011). The changing face of dentistry: nanotechnology. Int J Nanomedicine.

[29] Um CM, Ruyter IE (1991). Staining of resin-based veneering materials with coffee and tea. Quintessence Int.

[30] Poosti M, Ahrari F, Moosavi H, Najjaran H (2014). The effect of fractional CO2 laser irradiation on remineralization of enamel white spot lesions. Lasers Med Sci.

